# Diversity of Middle East respiratory syndrome coronaviruses in 109 dromedary camels based on full-genome sequencing, Abu Dhabi, United Arab Emirates

**DOI:** 10.1038/emi.2017.89

**Published:** 2017-11-08

**Authors:** Mohammed Farouk Yusof, Krista Queen, Yassir Mohammed Eltahir, Clinton R Paden, Zulaikha Mohamed Abdel Hameed Al Hammadi, Ying Tao, Yan Li, Abdelmalik Ibrahim Khalafalla, Mang Shi, Jing Zhang, Muzammil Sayed Ahmed Elhaj Mohamed, Mahmud Hamed Abd Elaal Ahmed, Ihsaan Abdulwahab Azeez, Oum Keltoum Bensalah, Ziyada Swar Eldahab, Farida Ismail Al Hosani, Susan I Gerber, Aron J Hall, Suxiang Tong, Salama Suhail Al Muhairi

**Affiliations:** 1 Abu Dhabi Food Control Authority, Abu Dhabi, UAE; 2 Division of Viral Diseases, National Center for Immunizations and Respiratory Diseases, Centers for Disease Control and Prevention, Atlanta, GA, USA; 3 Oak Ridge Associated Universities Fellow, Oak Ridge, TN, USA; 4 University of Sydney, Sydney, NSW, Australia; 5 IHRC Inc., Atlanta, GA, USA; 6 Abu Dhabi Food Control Authority, Al Ain, UAE; 7 Health Authority of Abu Dhabi, Abu Dhabi, UAE

**Keywords:** dromedary, MERS, Middle East respiratory syndrome coronavirus, United Arab Emirates

## Abstract

Middle East respiratory syndrome coronavirus (MERS-CoV) was identified on the Arabian Peninsula in 2012 and is still causing cases and outbreaks in the Middle East. When MERS-CoV was first identified, the closest related virus was in bats; however, it has since been recognized that dromedary camels serve as a virus reservoir and potential source for human infections. A total of 376 camels were screened for MERS-Cov at a live animal market in the Eastern Region of the Emirate of Abu Dhabi, UAE. In all, 109 MERS-CoV-positive camels were detected in week 1, and a subset of positive camels were sampled again weeks 3 through 6. A total of 126 full and 3 nearly full genomes were obtained from 139 samples. Spike gene sequences were obtained from 5 of the 10 remaining samples. The camel MERS-CoV genomes from this study represent 3 known and 2 potentially new lineages within clade B. Within lineages, diversity of camel and human MERS-CoV sequences are intermixed. We identified sequences from market camels nearly identical to the previously reported 2015 German case who visited the market during his incubation period. We described 10 recombination events in the camel samples. The most frequent recombination breakpoint was the junctions between ORF1b and S. Evidence suggests MERS-CoV infection in humans results from continued introductions of distinct MERS-CoV lineages from camels. This hypothesis is supported by the camel MERS-CoV genomes sequenced in this study. Our study expands the known repertoire of camel MERS-CoVs circulating on the Arabian Peninsula.

## INTRODUCTION

Middle East respiratory syndrome coronavirus (MERS-CoV) was first identified in the Kingdom of Saudi Arabia in October 2012 in a patient with acute respiratory distress syndrome.^1^ Later, a hospital-associated outbreak of respiratory illness in Jordan from April 2012 was found to be caused by MERS-CoV after retrospective testing of samples saved from case-patients.^2^ As of 10 August 2017, there have been 2040 laboratory-confirmed cases of infection with MERS-CoV from 27 countries reported to the World Health Organization with a reported case fatality ratio of 34.9%.

Initially after MERS-CoV was identified, bats along with a wide range of other animals common to the geographic areas where human MERS cases were detected were tested extensively for MERS-CoV. Evidence of MERS-CoV transmission between dromedary camels (camels) and humans was initially provided through two separate epidemiologic investigations of human MERS cases in Qatar and Saudi Arabia.^3, 4^ In these cases, camels that had been in contact with the human cases just before their illness onset were tested and found to be positive for a genetically very similar or identical MERS-CoV as that identified in the human cases. A large serological study performed in Saudi Arabia beginning in December 2012 provided additional evidence suggesting increased risk of MERS-CoV infection among people with frequent exposure to camels. The authors reported that 0.15% of individuals in the general population were positive for antibodies to MERS-CoV, but those with camel-exposure (e.g., slaughterhouse workers) had significantly higher seropositivity, 3.6%.^5^ Numerous different camel sample types have been investigated for virological confirmation of MERS-CoV RNA, including nasal swabs and secretions, oral swabs, rectal swabs, blood, feces and milk; the presence of MERS-CoV RNA has most consistently been detected in nasal swabs from infected camels.^3, 6^ Younger camels (aged <2 years) are more likely to be MERS-CoV RNA-positive and have higher viral loads compared with older camels.^7^ Serological samples from as early as the 1980s show that camels in the Middle East and northern Africa have antibodies to MERS-CoV or a very similar virus.^8, 9, 10^ These seroprevalence results do not hold true for Bactrian camels or for dromedaries from other regions such as Australia or Kazakhstan.^11, 12^ While these studies support the notion that camels can be a source of human infections, camels may not be solely responsible for human MERS cases as primary cases sometimes report no camel contact. Furthermore, the receptor for MERS-CoV, dipeptidyl peptidase 4, is fairly conserved in mammals, suggesting potential roles for other mammalian hosts.^13^

Additional work has been focused on understanding the course of infection in camels and related species. Experimental infection of camels leads to mild clinical illness and high loads of viral shedding from nasal discharge.^14^ Analysis of the upper respiratory tracts of camels has shown the presence of the MERS-CoV receptor, dipeptidyl peptidase 4 expressed in respiratory epithelial cells.^14^ These experimental results were similar to what has been documented in natural infection of camels with MERS-CoV.^8, 15, 16^ Alpacas, due to their close genetic relationship to camels, have also been shown under natural settings to be seropositive for MERS-CoV^17^ and under experimental settings were able to be infected with and shed MERS-CoV.^18^

Since the first human MERS-CoV was sequenced, studies have focused on sequencing and classifying MERS-CoVs in both humans and camels.^19, 20, 21^ Sabir *et al.*^21^ sequenced and analyzed representative MERS-CoV genomes, including 67 from camels, from regions within Saudi Arabia and found distinct lineages of clade B MERS-CoV. Additional non-MERS-CoVs were also detected, and co-infections with MERS-CoV and other coronaviruses was common. Another study sequenced 10 different camel MERS-CoVs from Abu Dhabi and found viruses in several lineages in clade B, including a sixth clade B lineage as well as a camel MERS-CoV within clade A.^20^ This study provided interesting insight into camel MERS-CoV diversity in nature as it is thought that the clade A MERS-CoVs are older and not circulating currently.^20^ Additional efforts for sequencing MERS-CoV from camels have focused on sequencing certain genes such as nucleocapsid or spike as potential spots for identifying genetic variation.^7, 22, 23^

Several of the human coronaviruses are thought to be of zoonotic origin. Human coronaviruses severe acute respiratory syndrom, 229E and NL63 are thought to have originated from bat coronaviruses.^24, 25^ OC43 is also thought to be of animal origin, with evidence suggesting a recent zoonotic transmission event.^26^ Recently, detection of a MERS-like coronavirus in a Ugandan bat implicated bats as the evolutionary source of MERS-CoV.^27^ The large RNA genome of coronaviruses is amenable to recombination both within closely related CoVs and between different CoVs.^28, 29, 30^ Circulating human CoVs have demonstrated the propensity to recombine and form new genotypes (e.g., OC43, genotypes D and E),^31, 32^ to recombine between different strains (NL63, Amsterdam 1 and 496 strains),^33, 34^ or to recombine with other animal CoVs (HKU1 is able to recombine with murine hepatitis virus at various nonstructural genes).^35^ Severe acute respiratory syndrom-CoV, which caused a pandemic in 2002–2003, is hypothesized to have undergone recombination before transmission in humans.^36^ A recent study found recombination signatures that delineate five major lineages with the recent MERS-CoV from the Republic of Korea outbreak originating from recombination events between lineages 3 and 4.^21^

The United Arab Emirates has reported numerous outbreaks of MERS-CoV since the virus was identified and has had the third highest number of confirmed human cases behind Saudi Arabia and South Korea.^37^ Previous testing of 7803 camels from February to March 2014 from zoos, public escorts, slaughterhouses and the borders with Saudi Arabia and Oman performed in the Emirate of Abu Dhabi showed that the highest MERS-CoV positivity rate among camels was seen at slaughterhouses, 8.25%, with an overall PCR positive rate of 1.6%.^38^ During 2013–2014 in the Emirate of Abu Dhabi, six clusters of human-to-human transmission were identified and analyzed in health-care outbreaks, household-associated clusters and sporadic cases.^39, 40^ Another study demonstrated the close relationship of the virus in camels and the introduction of the virus in human clusters^23^ and clusters associated with health-care facilities. The market sampled in this study has previously been linked to a human case in February 2015, a German traveler that was infected with MERS-CoV after visiting a market in the eastern region and subsequently died from complications of the infection.^41^ In this paper, we address the need for large-scale sequencing efforts to better understand the diversity of MERS-CoVs present within camel populations at a livestock market in Abu Dhabi, UAE.

## MATERIALS AND METHODS

### Camel samples

This study was carried out in a livestock market located within the eastern region of Abu Dhabi Emirate, UAE, during the period of March–April 2015 at Abu Dhabi Food Control Authority Veterinary Laboratory Abu Dhabi, UAE in response to the MERS-CoV notification from the Health Authority of Abu Dhabi of a German citizen who visited the same livestock market during his travels in UAE. Collection of samples from camels performed in this study was approved by the Centers for Disease Control and Prevention (CDC) Institutional Animal Care and Use Committee protocol number 2702HALMULX. A total of 376 nasal swabs were collected during week 1 from a livestock market and screened for MERS-CoV by PCR. These included 210 dromedaries that originated from local farms within Abu Dhabi Emirate, 106 camels from Oman and 60 camels of unknown origin. Camels sampled were predominantly male (*n*=269, 71.5%) and aged <1 year (*n*=255, 67.8%). After week 1, positive camels from pens 17, 19 and 20 were microchipped and sampled in weeks 3, 4, 5 and 6.

### Extraction and MERS-CoV PCR

Total RNA was extracted and purified using the EZ1 Virus Mini Kit 2.0 (Qiagen, Hilden, Germany). For screening, primers and probes from LightMix Modular MERS-CoV upE (Molbiol, Berlin, Germany) were used, one-step quantitative PCR with reverse transcription amplification and detection was done locally at Abu Dhabi Food Control Authority, Al Ain, UAE on a LightCycler 2.0 with LightCycler RNA Virus Master Chemistry from Roche (Basel, Switzerland). Samples positive by the upE assay were then subjected to the confirmation PCR assay using the LightMix Modular MERS-CoV Orf1a (Molbiol). The extracted RNA that was positive by MERS-CoV PCR was sent to the CDC, USA for sequence analysis.

### Fluidigm access array PCR and MiSeq amplicon sequencing

Overlapping primer pairs that span the entire MERS-CoV genome were developed and used in conjunction with the Fluidigm Access Array system (San Francisco, CA, USA). The Fluidigm Access Array was used to set up and perform one-step PCR with reverse transcription on 48 wells (15 samples with triplicate per sample) × 48 PCRs with reverse transcription according to the manufacturer’s protocol, allowing the ability to amplify and sequence multiple full genomes simultaneously. Resulting amplicon pools from each sample were sheared from 800–1200 bp to 200–300 bp using a Covaris M220 sonicator (Woburn, MA, USA). Sheared DNA from the PCR amplification of each camel sample was used to generate barcoded libraries for multiplexed sequencing using the NEBNext Ultra II DNA library prep kit (New England Biolabs, Ipswich, MA, USA). Sequencing was performed using an Illumina MiSeq instrument (San Diego, CA, USA).

Next-generation sequencing data were analyzed using a custom workflow in CLC Genomics Workbench 8.5. Sequencing adapters were trimmed, then 26 bp were trimmed from each end to remove any residual PCR primer sequence. Remaining reads were trimmed from the 3′-end using a CLC cumulative quality score of 0.05. Trimmed reads were aligned to a reference, either Jordan-N3/2012 (KC776174.1) or AbuDhabi_UAE_8 (KP209306.1). Consensus sequence was called based on regions that had 10 × or greater coverage. Variants were called using the CLC consensus caller with cutoff values of 5, 10, 15 and 35%, with a minimum count of five reads. Further analysis of these sequences was performed with custom scripts written in-house at the CDC.

### Fill in PCR and Sanger sequencing analysis

Thirty-two pairs of nested PCR assays that span the genome were designed based on alignment of available MERS-CoV genomes.^39, 40^ MERS-CoV genomes or partial genomes were amplified as needed to fill the gaps missed by Fluidigm Access Array PCR. Positive bands of the expected size that had strong signal and without additional bands were cleaned up using Exonuclease I (New England Biolabs) and Shrimp Alkaline Phosphatase (Roche). Samples were incubated at 37 °C for 15 min followed by 80 °C for 15 min to inactivate the Exonuclease and Shrimp Alkaline Phosphatase. Purified PCR amplicons were sequenced with the PCR primers in both directions on an ABI Prism 3130 Automated Capillary Sequencer (Applied Biosystems, Foster City, CA, USA) using Big Dye 3.1 cycle sequencing kits (Life Technologies, Carlsbad, CA, USA). The Sanger sequence data were analyzed using Sequencher 5.0. Ends and low-quality regions were trimmed manually. Contigs and consensus sequences were generated.

### Phylogenetic analysis

The nucleotide sequences were first aligned in MAFFT v7.013^42^ with published MERS-CoV sequences retrieved from GenBank. Phylogenetic trees were then inferred using the maximum likelihood method available PhyML version 3.0^43^ assuming a general time-reversible model with a discrete gamma distributed rate variation among sites (Gamma_4_) and a SPR tree swapping algorithm and visualized using MEGA version 6.^44^

### Recombination analysis

The initial detection of recombination was performed based on the full-genome alignment used for phylogenetic analyses using representative MERS-CoV genomes from NCBI and the full-genome sequences from this study. To detect the potential recombination sequences within the data set, we used RDP,^45^ GENECOV,^46^ Chimera^47^ and 3Seq^48^ methods implemented in the program RDP v4.16.^49^ Default settings were used for each detection methods, and only events detected by two or more methods were considered recombination. The RDP program also provided the location information of possible recombination breakpoints, which were then confirmed and/or further examined using the Distant Plot program implemented in RDP. In order to identify potential parental strains/types associated with each recombination event, the alignment was separated based on the breakpoints into non-recombined regions, each subject to phylogenetic analyses for parental strains/types identification.

### Average nucleotide differences

The number of base differences per lineage and within lineage from averaging overall sequence pairs between groups were determined in MEGA6. The analysis involved 254 nucleotide sequences. Codon positions included were 1st+2nd+3rd+Noncoding. All positions containing gaps and missing data were eliminated. There were a total of 25 193 positions in the final data set. Evolutionary analyses were conducted in MEGA6.^44^

## RESULTS

### MERS-CoV detection in camels and MERS-CoV phylogenetic analysis

In all, 376 camels were screened by MERS-CoV upE PCR and a total of 109 camels were positive (29%) during week 1 of the study, characteristics of the positive camels are listed in Table 1. A total of 16 camels that were positive in week 1 were monitored and tested weekly in weeks 3 through 6, and an additional 30 nasal swabs were positive. From 139 total positive samples, full-genome sequences were obtained from 126 samples and nearly full genomes, missing 4 kilobases or less, were obtained from 3 samples for a total of 129 full or nearly full camel MERS-CoV genomes. In addition, alternate PCR strategies were used to sequence the S genes for the 10 samples from which we were unable to obtain full genomes due to low viral load. Full or nearly full S genes were obtained from 5 of those 10 samples. All sequences have been deposited in GenBank, accession numbers MF598594-MF598722 for the full-genome sequences and MF679171-MF679174 for S gene sequences.

The sequences of these 129 camel MERS-CoV genomes shared >99% identity compared to previously published MERS-CoV sequences. We also compared amino-acid variations in the Spike protein between human and camel MERS-CoVs, which contains the receptor-binding domain (residues 358–588) that is responsible for mediating attachment with the MERS-CoV receptor, dipeptidyl peptidase 4. Across multiple alignments of Spike proteins using a 50 amino-acid window (Supplementary Figure 1), we observed that camel- and human-derived MERS-CoV Spike sequences both have amino-acid variations throughout, although overall camel-derived sequences show slightly more variations. However, we observed no significant or unique amino-acid changes present between human and camel MERS-CoV Spike proteins. In addition, genome analysis of the full or near-full-genome sequences identified similar open reading frames (ORFs) and genome structure as compared to the known MERS-CoV except viruses MF598715, MF598719, MF598720, MF598721 and MF598722 that all contain a truncated NS4B of 99 amino acids versus the regular length of 247 amino acids and another virus (MF598690) with a truncated NS4B of 92 amino acids, all due to an early stop codon mutation.

**Figure 1:**
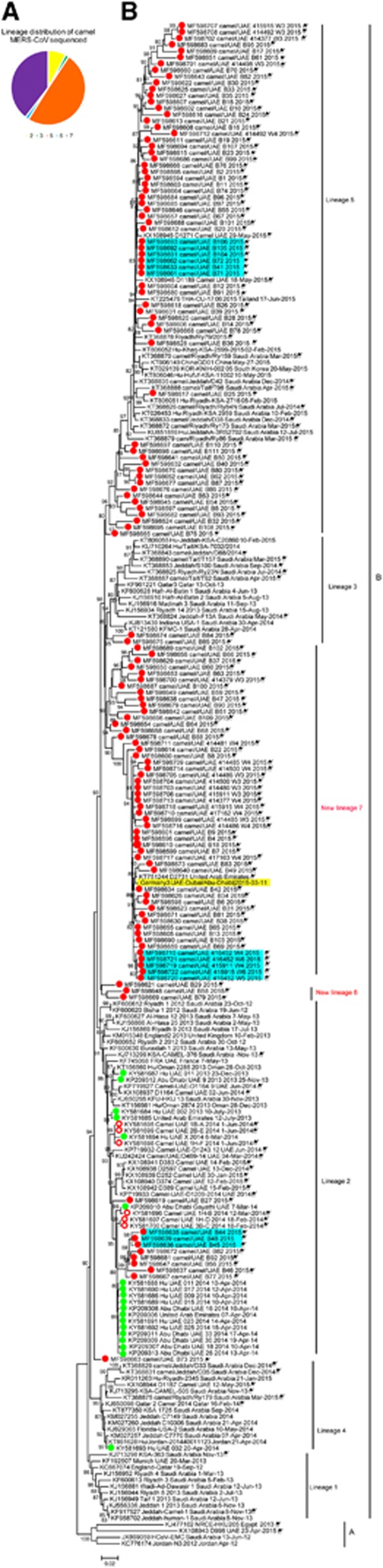
Phylogeny of MERS-CoV full genomes or near-full genomes from humans and dromedary camels. (**A**) The number of samples sequenced in this study that fell into each lineage. Yellow, lineage 2; green, lineage 3; orange, lineage 5; blue, lineage 6; and purple, lineage 7. (**B**) Maximum likelihood phylogenetic analysis of 126 complete and 3 nearly complete camel MERS-CoV genomes and 111 previously published human and camel MERS-CoV genomes. Red closed circles, camel samples sequenced in this study. Red open circles, camel samples previously sequenced from UAE by our lab. Green closed circles, human samples previously sequenced by our lab. Camel samples are denoted with a camel symbol. * indicate genomes that are nearly complete. Yellow shading indicates the German MERS-CoV case linked to the market in this study. Light blue shading indicates closely related sequences from camels that were housed in the same pen. Bootstrap values are shown next to the branches. The scale bar indicates the number of nucleotide substitutions per site. Middle East respiratory syndrome coronavirus, MERS-CoV.

Maximum likelihood phylogenetic analysis was performed on 240 MERS-CoV genomes including 129 full or nearly full MERS-CoV genomes generated in this study (Figure 1); lineages are labeled according to the naming system used previously.^21^ The camel MERS-CoVs sequenced in this study were placed throughout clade B of the MERS-CoV tree. A majority of the viruses sequenced in this study, 64 (50%) of the camel MERS-CoV genomes (Figures 1A and 1B), fell within the established lineage 5 with human MERS-CoV sequences from the Republic of Korea outbreak and including some that are closely related to human MERS-CoVs. Of these, camel MERS-CoV genome MF598617 is only 15 nucleotides different than a human MERS case from Riyadh, Saudi Arabia in 2015 (KT806051). In addition, camel MERS-CoV genome MF598618 is 30 nucleotides different than a human MERS case identified in Thailand from an Omani national (KT225476). Both of these camel MERS-CoV genomes are more closely related to the human cases than previously identified camel MERS-CoVs. Ten (8%) of the camel MERS-CoV genomes analyzed fell within lineage 2 along with other previously sequenced camel MERS-CoVs and clusters of human MERS-CoV sequences from UAE. Eight of these camel MERS-CoV sequences formed a monophyletic group with each other within lineage 2 and are closer to the human MERS-CoVs from the 2014 UAE outbreak cluster than to other human MERS-CoVs in lineage 2.^50^ Two (1.5%) camel MERS-CoV sequences fell within lineage 3. Lineage 3 is comprised of a number of human and camel MERS-CoVs from Saudi Arabia and Qatar. Overall, the lineage distribution of camel MERS-CoVs sequenced in this study was throughout the different pens sampled (Supplementary Table 1).

The average number of nucleotide changes between lineages and within lineages was determined with the previously published lineages and new lineages (Supplementary Tables 2 and 3). The lowest average number of nucleotide changes between distinct lineages was 39.3995 and within lineages the average number of nucleotide changes ranged between 15.786 and 27.289. We identified two potentially new lineages (6 and 7) of MERS-CoV within clade B that are distinct from previously identified lineages.^21^ Lineage 6 (*n*=2, 1.5%) contains two camel MERS-CoV sequences and does not have any human MERS-CoVs identified yet. Lineage 6 has an average number of nucleotide changes compared to previously identified lineages ranging from 36.031 to 46.545, which is near the threshold of nucleotide changes needed for consideration as a new lineage. As the topological support for lineage 6 is low, its placement in the phylogeny should be treated with caution. Forty percent (*n*=51) of the camel MERS-CoV genomes we sequenced fell within the other new lineage (proposed name, lineage 7). Lineage 7 has an average number of nucleotide changes 43.763–60.25 from the other previously published lineages, well above the lowest average nucleotide changes between known lineages of 39.399 (Supplementary Table 2). Lineage 7 is comprised of camel MERS-CoVs and one human MERS-CoV, an imported case in Germany from the United Arab Emirates.^41^ The camel MERS-CoVs most closely related to the German case (highlighted in yellow, Figure 1B) had just 7 and 20 nucleotide differences (MF598634 and MF598673, respectively) compared to the MERS-CoV sequence from the German patient. In the phylogeny, two additional genomes did not fall within established lineages and their phylogenetic positions were not well-supported, MF598621 and MF598663. For camel genome MF598621 (Figure 1 and Supplementary Table 2), the average nucleotide differences range was 28.266–42.818, with the closest similarity being with lineage 2 viruses. Camel genome MF598663 had an average nucleotide change of 27.728 nucleotides compared with lineage 5 viruses, suggesting a closer relationship with lineage 5 viruses. Finally, none of the camel MERS-CoV genomes sequenced in this study fell within clade A or lineages 1 or 4 of clade B.

### Camels sampled multiple times

**Figure 2:**
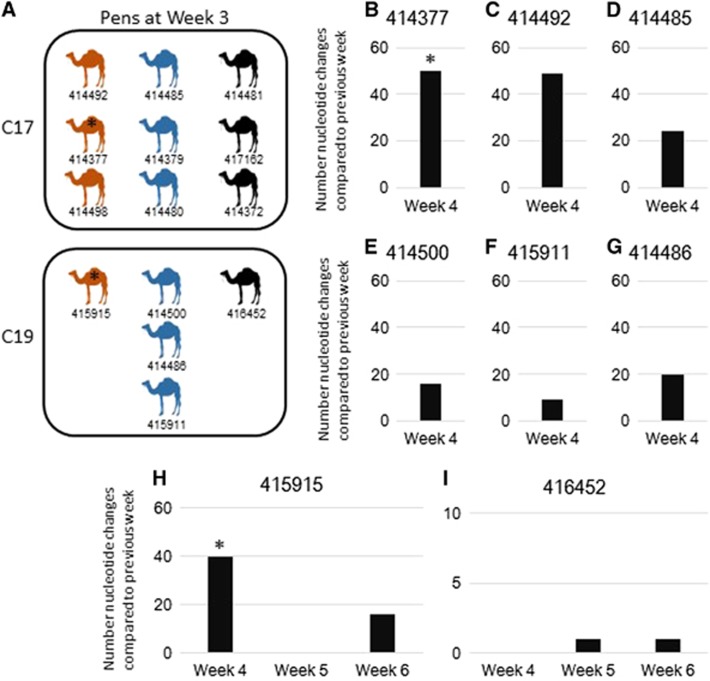
Analysis of camels sampled multiple weeks. (**A**) Schematic representation of camels housed together. Camel numbers are listed below each camel. Orange camels, camels with lineage 5 virus in week 3; blue camels, camels with lineage 7 virus in week 3; black camels, camels that we were unable to obtain a full-genome sequence from in week 3. (**B**–**I**) Nucleotide changes in camels sampled multiple times. x axis, week sampled and camel sampled; y axis, number of nucleotide changes compared to previous week sampled. Number of changes are relative to the previous genome sequenced for each camel. * represents camel sample pairs that changed lineages between the weeks compared. (**B**) Camel 414377 MF598702/MF598713 (week 3/4). (**C**) Camel 414492 MF598708/MF598712 (week 3/4). (**D**) Camel 414485 MF598699/MF598709 (week 3/4). (**E**) Camel 414500 MF598704/MF598714 (week 3/4). (**F**) Camel 415911 MF598706/MF598719 (week 3/4). (**G**) Camel 414486 MF598705/MF598716 (week 3/4). (**H**) Camel 415915 MF598707/MF598718/MF598722 (week 3/4/6). (**I**) Camel 416452 MF598715/MF598720/MF598721 (week 4/5/6).

To understand the stability of MERS-CoV infection in camels, positive camels from pens C17, C19 and C20 were identified via microchip and sampled weekly during weeks 3–6 of this study. Figure 2A shows a housing schematic of these camels sampled multiple times, note pen C20 is not shown as no full genomes were obtained from samples from pen C20. Both pens C17 and C19 housed a mixture of camels that had MERS-CoV from lineages 5 and 7 based on the sequencing in week 3. Of the eight sets of samples which, had full or near-full-genome sequences available for comparison, camels 414377 (Figure 2B) and 414492 (Figure 2C) were sampled weeks 3 and 4 and had full genomes available for comparison with 50 and 49 nucleotide changes between the MERS-CoV genomes compared, respectively. For camel 414377, this represented a change of MERS-CoV lineage: lineage 5 in week 3 and lineage 7 in week 4. Of note, camel 414377 sampled in week 4 had identical sequence to camel 414480 that was housed in the same pen (C17, Figure 2A). Camels 414485 (Figure 2D), 414500 (Figure 2E) and 415911 (Figure 2F) were sampled weeks 3 and 4 and had between 9 and 24 nucleotide changes between weeks 3 and 4, which did not represent any changes in lineage. Camels 414486 (Figure 2G), 415915 (Figure 2H) and 416452 (Figure 2I) were sampled during weeks 3, 4, 5 and 6. Camel 414486 had 20 nucleotide changes between weeks 3 and 4 and was negative for MERS-CoV after week 4 (Figure 2G). Camel 415915 had 40 nucleotide changes, which represented a lineage switch from lineage 5 to lineage 7 during week 4 sampling (Figure 2H). The sequence of camel 415915 at week 4 had only 5 nucleotide differences compared to camels 415911 and 414500 from lineage 7, which were also housed in the same pen. Camel 415915 was negative week 5 but positive again week 6 and had 16 nucleotide changes compared to week 4 with no change in lineage. Camel 416452 (Figure 2I) had samples available for comparison from weeks 4 through 6, as the full-genome sequence was not available for week 3. Camel 416452 had 1 nucleotide change between week 4 and 5 and 1 nucleotide change between week 5 and 6.

### Frequent recombination identified in camels

**Figure 3:**
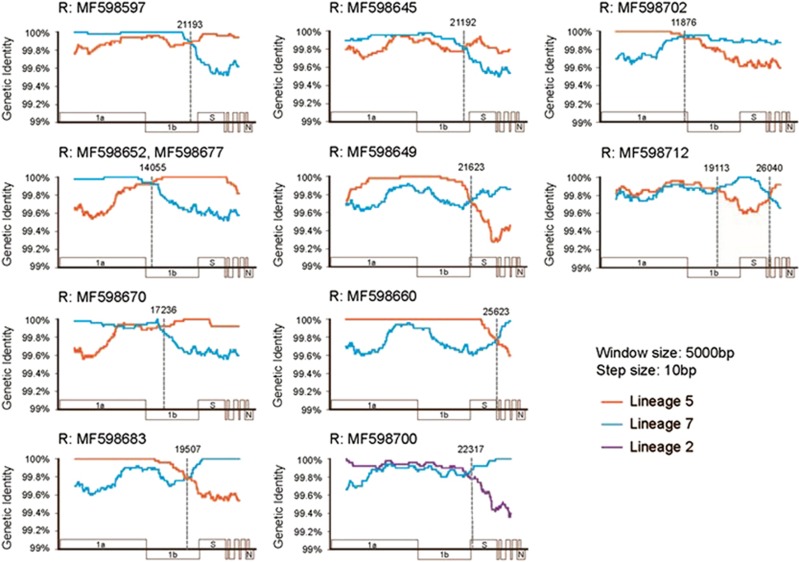
Characterization of recombination breakpoints and parental types. Each panel shows the similarity plot of a single recombination event. The different colored lines represent the similarity comparisons of the recombinant to its two potential parental strains from different lineages: orange, lineage 5; blue, lineage 7; and purple, lineage 2. The gray dotted line marks the potential location of recombination breakpoints.

Recombination analysis was performed on the camel MERS-CoV genomes sequenced in this study to determine whether any recombination events occurred. A total of 10 well-supported recent recombination events were detected (Table 2), which involved 11 viruses, and the percentage of detection from this data set is thus 8.5%. Among these, 9 recombination events have single breakpoints and 1 has double breakpoints. The most common location for these breakpoints was around the ORF1b/S gene junction (Table 2). While most of the recombination events were represented by a single virus, one recombination event that occurred involved two viruses, MF598652and MF598677, which were nearly identical (one nucleotide difference) and were apparently derived from a single recombinant origin. The analysis of parental groups revealed that most of the recombination events were between lineages 5 and 7 (Table 2 and Figure 3); whereas, a single event occurred between lineages 2 and 7 (Table 2 and Figure 3, MF598700).

### Minor variants in camel MERS-CoVs

**Figure 4:**
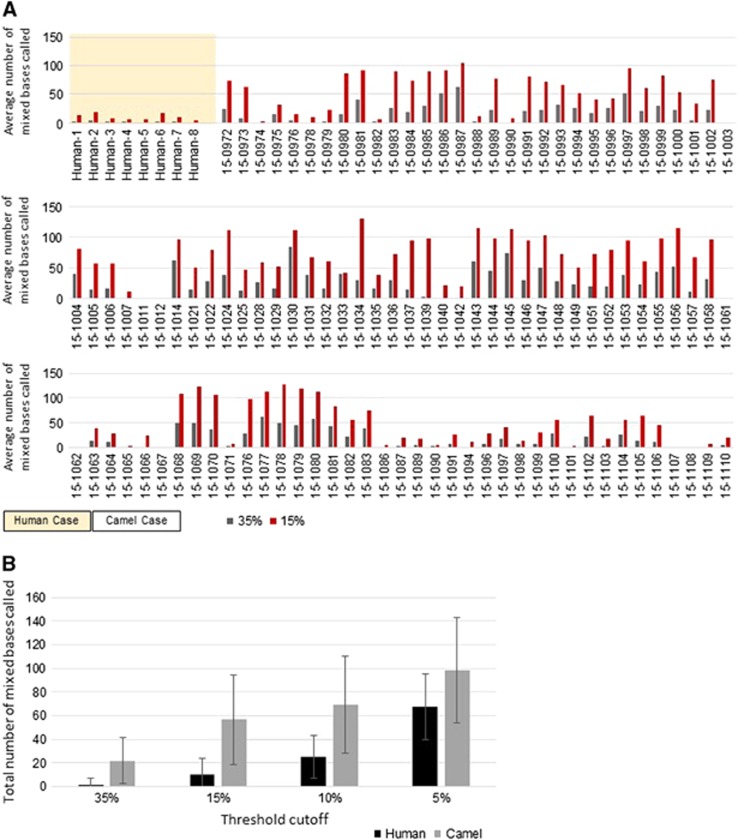
Minor variant analysis. (**A**) Average number of mixed bases called in human and camel MERS-CoV genomes. Gray bars, 35% threshold cutoff; red bars, 15% threshold cutoff. (**B**) Total number of mixed bases called at varying threshold levels between humans (black) and camels (gray). Middle East respiratory syndrome coronavirus, MERS-CoV.

In order to understand the diversity of the MERS-CoV variants within individual camels, we generated consensus sequences from next generation sequencing data, incorporating variability data at each site. We also used next generation sequencing data from a limited number of available primary human MERS samples that had been amplified and sequenced in a similar manner to the camel MERS-CoVs in this study, six of which came from human MERS cases in UAE.^50^ For each sample, mixed bases were called, with cutoff thresholds of 5, 10, 15 and 35% of high-quality read coverage at each base. The minor variants at 15 and 35% thresholds were quantified for each individual camel or human MERS-CoV genome and plotted as the total number of minor variants at each cutoff (Figure 4A). The data from each cutoff was averaged and plotted as an average number of mixed bases for camels or for humans (Figure 4B). In general, there were more minor variants found in the camel samples than for the human samples. There were an average of 1.67 (at 35% threshold) variants per sample and 10.5 (at 15% threshold) variants per sample from the human-derived viruses, while there was an average of 20.6 and 53.6 (35% and 15% thresholds, respectively) variants per sample for the camel viruses.

## DISCUSSION

In the present study, MERS-CoV RNA was detected in 109 (29%) out of the 376 camels screened at a livestock market in Abu Dhabi Emirate, UAE sampled between March and April 2015. Such prevalence is higher compared to the previously reported 1.6–15% molecular prevalence detected in camels at the borders with Saudi Arabia and Oman, slaughterhouses and farms, suggesting that livestock markets may represent a location for greater potential for human and camel infections.^23, 38, 51^ A total of 129 full or nearly full MERS-CoV genomes were obtained from camels sampled in this study, including camel MERS-CoV genomes that are more closely related to human MERS cases than previously available camel MERS-CoV genomes. Within lineage 5, two camel MERS-CoV genomes, MF598617 and MF598618, were more related to human MERS cases (KT806051 and KT225476, respectively) than previous camel MERS-CoVs, with only 15 nucleotide differences between MF598617 and KT806051 (differences were throughout the genome, with 1 difference in the spike gene) and 30 nucleotide differences between MF598618 and KT225476 (differences were primarily in ORF1AB and spike genes). Interestingly, while the camels sampled in this study came from UAE and Oman, the human MERS-CoV genome (KT806051) related to MF598617 was from a case in Riyadh, Saudi Arabia. In addition, a camel MERS-CoV that is closely related to the 2015 Germany imported case from UAE was identified with only 7 nucleotide differences, 2 of which were in the spike gene. A previously sequenced camel MERS-CoV genome was also only 7 non-overlapping nucleotides different than the 2015 Germany imported case, but that sequence was obtained from a camel in a closed dairy herd in Dubai (KT751244). This study demonstrates that livestock markets may actively participate in the spread of MERS-CoV among camels and/or humans. Unlike the severe acute respiratory syndrom-CoV outbreak, which originated from a limited number of zoonotic events and then acquired characteristic genetic changes associated with more efficient person-to-person spread, MERS-CoV cases and outbreaks appear to result at least in part from multiple ongoing zoonotic transmissions. The great diversity of MERS-CoVs in humans is mirrored in the camel population as shown in this study as well as previous studies.^8, 20, 21^ This supports the hypothesis that some human MERS-CoV infections are a result of multiple independent transmissions from camels.^8, 15^

Despite mounting evidence that camels can transmit MERS-CoV to humans, there have only been a few studies focused on full-genome sequencing analysis of MERS-CoVs from camels. In all, 129 full or nearly full-genome MERS-CoV sequences were obtained from 109 unique camels in this study. These camel MERS-CoV genomes sequenced were placed throughout the MERS-CoV phylogenetic tree and represented four lineages previously identified by Sabir *et al.*^21^ as well as two new lineages (lineages 6 and 7). The majority of samples fell within the established lineage 5 and the lineage 7. Lineage 5 MERS-CoV sequences include human MERS-CoVs from the Republic of Korea outbreak as well as human cases from Saudi Arabia.^52^ Previously, Lau *et al.*^20^ identified two human MERS-CoV sequences, KF600612 and KF600620, both from Saudi Arabia from 2012, as a potentially new lineage. Sabir *et al.*^21^ placed these two sequences as an outlier to lineage 2 and our analysis places them within lineage 2. Lineage 7 is almost entirely comprised of MERS-CoVs genomes from camels, with the notable exception of the travel-related MERS-CoV infection in a German citizen who visited a livestock market in UAE before becoming ill. We also detected smaller numbers of camel MERS-CoVs within two known lineages (2 and 3) and a new lineage, 6. Interestingly, in three separate pens there were three separate instances of highly related MERS-CoV genomes identified indicating some of the camels may have been infected at the market by their pen mates. Most of the different lineages of MERS-CoV identified were throughout the pens and of the 32 pens sampled, 16 had more than one lineage of MERS-CoV present. This large diversity of camel MERS-CoVs was detected in camels sampled from a single livestock market. This is in contrast to a large sequencing study, which sequenced camels from several cities and various locations camels were housed.^21^ In the previous study, camels were sampled from slaughterhouses, farms and wholesale markets in Jeddah, Riyadh and Taif within Saudi Arabia. Despite a large sample size, our study did not detect any camel MERS-CoV sequences belonging to clade A, which has been identified in a camel from UAE in a previous study.^20^ A recent study of MERS-CoV in dromedaries in Egypt identified a potentially new MERS-CoV lineage, however only a limited amount of the Spike gene (600 bp) was sequenced and used for comparisons.^53^

In order to understand the dynamics of MERS-CoV infection in camels over time, previous studies have sampled camels at multiple time points and sequenced MERS-CoV. Samples collected from the same animals a month apart were genetically identical. In addition, adult camels showed evidence of reinfection with MERS-CoV; they were seropositive at the beginning of the study and later in the study MERS-CoV RNA was detected in nasal and fecal swabs.^16^ Another study also demonstrated the presence of MERS-CoV RNA in camel calves sampled at an 8-day interval although genetic comparisons were not performed on those samples.^54^ In contrast, in the camels sampled over multiple weeks from this study, there were significantly more nucleotide changes observed between 1-week intervals than would be expected based on the known mutation rate of MERS-CoV with lineage changes between 1-week intervals observed in two camels as well. This could be due to several reasons. First, sample tracking errors may have occurred. This is highly unlikely as the camels were microchipped after the first week and the microchips were used for camel identification between different weeks. Second, it is possible that these apparent differences stem from errors in sequencing. We have done extensive comparisons of our Fluidigm Access Array/MiSeq sequencing platform compared to the gold standard of Sanger sequencing. We use the same high-fidelity enzyme for both platforms and the Fluidigm Access Array platform has one round of PCR amplification, so it has a lower chance of introducing PCR bias. Finally, another reason for the higher than expected mutation rate could be due to the presence of co-infection or reinfection of camels with multiple MERS-CoVs present in the same pen. If this were the case, each week the predominant virus could change and thus we sequenced different viruses at different time points from the same animal as we observed in two camels sampled just 1 week apart. Given the extraordinarily high number of nucleotide changes within a 1-week timeframe for camels 414377, 414492 and 415915, it seems most likely that reinfection from camels within the same pen occurred. This was also supported by the fact that although they were from different origins, highly similar MERS-CoV were clustered in the same pen, suggesting transmission occurring within the same pen. For example, in the two camels that had MERS-CoVs of different lineages in different weeks (both lineage 5 to lineage 7), they were housed in pens and had identical or near-identical sequences with camels that had lineage 7 viruses also. Another camel sampled multiple times also demonstrated significant nucleotide changes (49) in a 1-week period. Although this did not reflect a lineage switch, it could also be due to reinfection by a different MERS-CoV from the same lineage in the same pen. Lineages are based on the topology of the phylogenetic tree with support from measuring the average number of nucleotide changes, in this instance this MERS-CoV genome was above the average. A longitudinal study in a fixed camel population such as a farm would be beneficial to further understand the dynamics of natural MERS-CoV infection in camels over time.

Coronaviruses, human and animal, are known for high recombination rates. Comparison of MERS-CoV and the highly related Neoromicia coronavirus (from a South African *Neoromicia capensis* bat) indicated that a nonrecent recombination event potentially gave rise to pathogenic MERS-CoV.^55^ Recent studies also support recombination of MERS-CoV in camels as well, including evidence that the MERS-CoVs recently circulating in the Republic of Korea and Saudi Arabia are of recombinant origin.^52, 56^ We were able to detect and describe 10 recombination events in the camel samples. One recombination event was detected in two samples, representing circulation of a common recombinant ancestor among camels. The junction between ORF1b and S was the most frequently identified location for the recombination breakpoints, which is in line with previous evidence in other coronaviruses, including a recent example between feline and canine alphacoronaviruses.^28, 57, 58, 59^ Expectedly, few recombination events were detected between more closely related viruses, i.e., within a lineage. It is important to take in to consideration that recombination between distantly related clades is much easier to detect than between closely related viruses, for which there were few phylogenetic signals. Therefore, it is highly likely that recombination events between more closely related viruses remain undetected.

Quasispecies have been identified in MERS-CoV genomes through deep sequencing.^60^ In this study, we observed a higher number of minor variants in camel-derived MERS-CoV genomes compared to the previously sequenced human-derived viruses. In this study, we kept the threshold for reporting minor variants relatively high to avoid calling sequencing errors or PCR artifacts as legitimate variants, so the data represent only a subset of the potential number of variants present. The data describe a larger, more diverse group of MERS-CoV variants or quasispecies present in camels than has been described in humans. This difference suggests that within that diverse repertoire in camels, there may be only a subset of viruses capable of being transmitted and replicating in humans. Understanding and detecting these variants will be critical in describing how MERS-CoV is transmitted both zoonotically and person to person. Indeed, this has been supported by the limited sequencing of camel MERS-CoV available and is now greatly expanded by our current study. The large population of MERS-CoV quasispecies within camels, coupled with the known circulation of other coronaviruses in camels and the recombination potential between coronaviruses could lead to highly divergent and novel coronaviruses.^20, 21, 28, 29, 30^

A One Health approach was the underlying principle on which this study was designed. It was known that a German citizen visited a livestock market that houses dromedaries and following that visit, was MERS-CoV-positive.^41^ Sampling and testing of camels at the market revealed a large diversity of MERS-CoVs circulating among camels. The genetic diversity of MERS-CoVs and the frequency of recombination suggest the potential for emergence of new MERS-CoV variants, which may be able to sustain more efficient person-to-person transmission, as with severe acute respiratory syndrom. This further underscores the importance of this and related future studies to characterize this diversity and guide development of targeted interventions.

## Figures and Tables

**Table 1:** MERS-CoV-positive camel samples March–April 2015

	**Week 1**	**Weeks 3–6**
*Gender*		
Male	73	14
Female	33	2
Unknown	3	0
		
*Origin*		
UAE	53	6
Oman	53	10
Unknown	3	0
		
*Age*		
<1 year	81	16
>1 year	25	0
Unknown	3	0
		
*Ct value (upE)*		
10 s	12	1
20 s	47	2
30 s	50	13

Abbreviation: Middle East respiratory syndrome coronavirus, MERS-CoV.

**Table 2:** Details of recombination events

**Event**	**Recombinant strain**	**Breakpoint**	**Parental A region**	**Parental A group**	**Parental A representative**	**Parental B region**	**Parental B group**	**Parental B representative**
**1**	MF598597	21193	1–21193	Lineage 7	MF598605	21994–30125	Lineage 5	MF598644
**2**	MF598652	14055	1–14055	Lineage 7	MF598689	14056–30125	Lineage 5	MF598625
	MF598677							
**3**	MF598670	17236	1–17236	Lineage 7	MF598689	17236–30125	Lineage 5	MF598611
**4**	MF598683	19507	1–19507	Lineage 5	MF598657	19508–30125	Lineage 7	MF598605
**5**	MF598645	21192	1–21192	Lineage 7	MF598630	21192–30125	Lineage 5	MF598615
**6**	MF598649	21623	1–21623	Lineage 5	MF598666	21623–30125	Lineage 7	MF598647
**7**	MF598660	25623	1–25623	Lineage 5	MF598633	25623–30125	Lineage 7	MF598629
**8**	MF598700	22317	1–22317	Lineage 2	MF598647	22317–30125	Lineage 7	MF598699
**9**	MF598702	11876	1–11876	Lineage 5	MF598657	11876–30125	Lineage 7	MF598605
**10**	MF598712	19113, 26040	1–19113, 26040–30125	Lineage 5	MF598666	19113–26040	Lineage 7	MF598709
