# Supplementary material for: Diversity of Middle East respiratory syndrome coronaviruses in 109 dromedary camels based on full-genome sequencing, Abu Dhabi, United Arab Emirates
Source: Emerg Microbes Infect. 2017 Nov 8;6(11):e101–. doi: 10.1038/emi.2017.89 (PMC5717090; doi:10.1038/emi.2017.89)
Supplement: Supplementary Tables [file emi201789x1.docx]

**Supplementary Table 1.** Lineage distribution in camel pens. Camel pens sampled are listed along with the lineage of MERS-CoV found within that pen. The number of camels that had that lineage virus is listed in parentheses after the lineage number.

**Supplementary Table 1.** Lineage distribution in camel pens

| Pen | Lineage (# camels) |
| --- | --- |
| 41 | 2 (1), 5 (2) |
| 42 | 2 (1), 5 (2) |
| 43 | 3 (1), 5 (1) |
| 46 | 3 (1) |
| 47 | 5 (1) |
| 49 | 5 (1) |
| 53 | 7 (2) |
| 54 | 2 (1) |
| 55 | 5 (1) |
| 56 | 7 (1) |
| 58 | 2 (3), 5 (1), 7 (3) |
| 76 | 2 (1), 5 (5), 6 (1), 7 (6) |
| 77 | 2 (1), 5 (20), 6 (1), 7 (10) |
| C17 | 5 (4), 7 (7) |
| C19 | 5 (1), 7 (12) |
| L42 | 5 (2) |
| L49 | 5 (1), 7 (1) |
| L50 | 7 (3) |
| L54 | 5 (1), 7 (1) |
| L55 | 5 (1) |
| L56 | 5 (1), 7 (1) |
| L57 | 5 (3) |
| L59 | 5 (2), 7 (1) |
| L74 | 5 (1) |
| L78 | 5 (2), 7 (1) |
| L80 | 5 (1), 7 (1) |
| L82 | 5 (1) |
| L85 | 2 (1) |
| L86 | 5 (1) |
| L90 | 2 (1), 5 (1) |
| L91 | 5 (1) |
| L94 | 5 (4), 7 (1) |
| Unknown | 5 (2) |

**Supplementary Table 2.** Average nucleotide changes between lineages. Lineages were labeled as groups in MEGA 6 and the average nucleotide changes between each lineage was computed. New lineages proposed in this study are indicated with an asterisk (*).

**Supplementary Table 2.** Average nucleotide changes between lineages

|  | | | |  |  |  |  |
| --- | --- | --- | --- | --- | --- | --- | --- |
|  | Lineage 1 | Lineage 2 | Lineage 3 | Lineage 4 | Lineage 5 | Lineage 6* | Lineage 7* |
|  |  |  |  |  |  |  |  |
| Lineage 2 | 54.009 |  |  |  |  |  |  |
| Lineage 3 | 52.505 | 45.613 |  |  |  |  |  |
| Lineage 4 | 38.482 | 51.602 | 49.413 |  |  |  |  |
| Lineage 5 | 49.141 | 49.893 | 39.399 | 42.177 |  |  |  |
| Lineage 6* | 46.545 | 36.031 | 40.6 | 40.375 | 37.457 |  |  |
| Lineage 7* | 60.25 | 49.593 | 45.784 | 52.807 | 43.763 |  |  |
| MF598621 | 42.818 | 28.266 | 38.55 | 35 | 33.228 | 29 | 37.887 |
| MF598663 | 35.273 | 30.266 | 37.25 | 29 | 27.728 | 29 | 40.774 |

**Supplementary Table 3.** Average nucleotide changes within lineages. Lineages were labeled as groups in MEGA 6 and the average nucleotide changes within each lineage was computed. New lineages proposed in this study are indicated with an asterisk (*).

**Supplementary Table 3.** Average nucleotide changes within lineages

| Lineage | Nucleotide changes | |
| --- | --- | --- |
| Lineage 1 | | 23.745 |
| Lineage 2 | | 27.289 |
| Lineage 3 | | 24.405 |
| Lineage 4 | | 15.786 |
| Lineage 5 | | 20.245 |
| Lineage 6* | | 26 |
| Lineage 7* | | 21.999 |
