# Supplementary figures and images for: Diversity of Middle East respiratory syndrome coronaviruses in 109 dromedary camels based on full-genome sequencing, Abu Dhabi, United Arab Emirates
Source: Emerg Microbes Infect. 2017 Nov 8;6(11):e101–. doi: 10.1038/emi.2017.89 (PMC5717090; doi:10.1038/emi.2017.89)

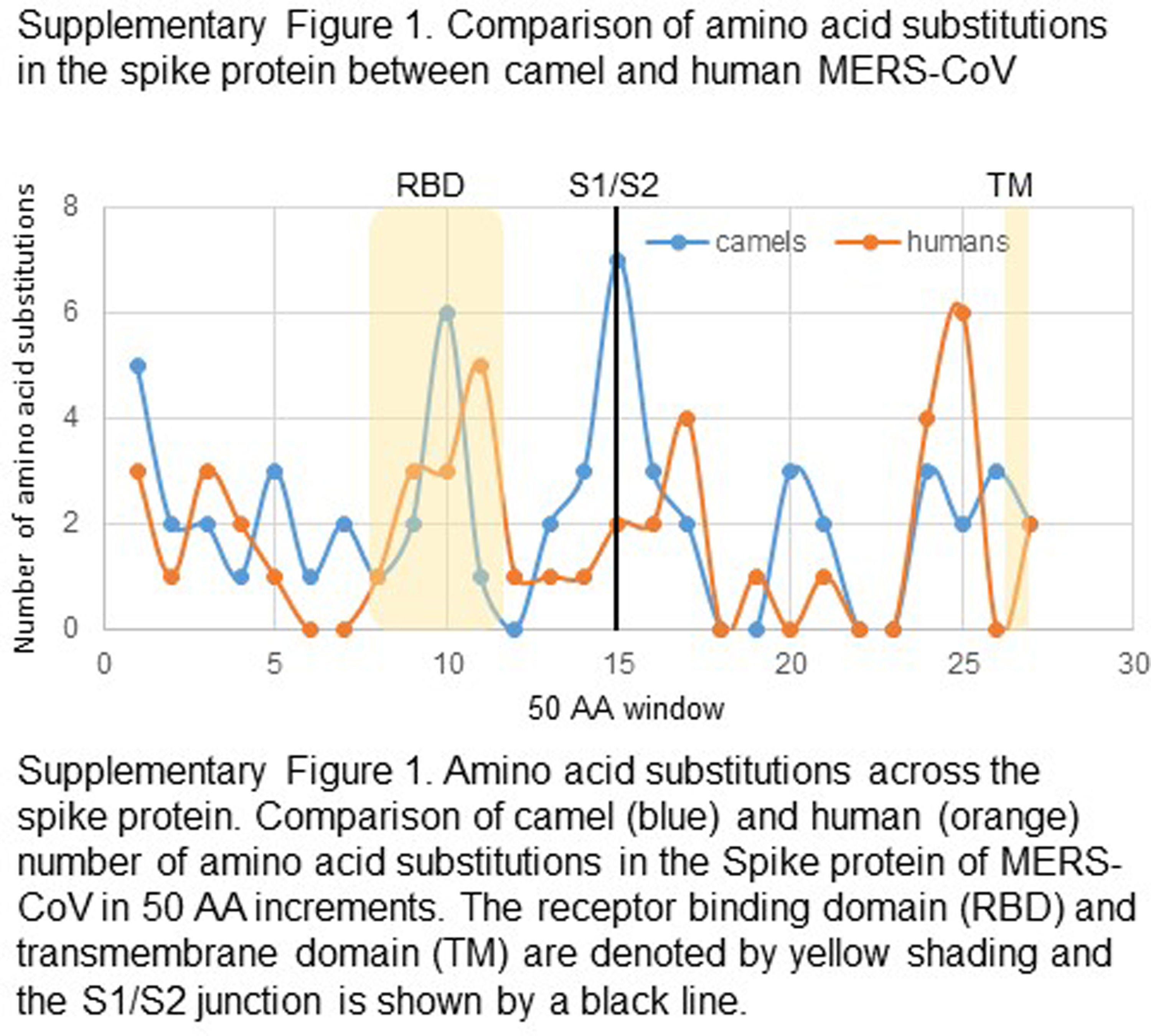

Supplement: Supplementary Figure 1 [file emi201789x2.tif]
